# Development of prediction models for lymph node metastasis in endometrioid endometrial carcinoma

**DOI:** 10.1038/s41416-020-0745-6

**Published:** 2020-02-10

**Authors:** Hege F. Berg, Zhenlin Ju, Madeleine Myrvold, Kristine E. Fasmer, Mari K. Halle, Erling A. Hoivik, Shannon N. Westin, Jone Trovik, Ingfrid S. Haldorsen, Gordon B. Mills, Camilla Krakstad, Henrica M. J. Werner

**Affiliations:** 1 Centre for Cancer Biomarkers; Department of Clinical Science, University of Bergen, Bergen, Norway; 2 Department of Obstetrics and Gynaecology, Haukeland University Hospital, Bergen, Norway; 3 Bioinformatics and Computational Biology, UT M.D. Anderson Cancer Center, Houston, TX USA; 4 Section for Radiology, Department of Clinical Medicine, University of Bergen, Bergen, Norway; 5 Department of Radiology, Haukeland University Hospital, Bergen, Norway; 6 Department of Gynaecologic Oncology and Reproductive Medicine, UT M.D. Anderson Cancer Center, Houston, TX USA; 7 Department of Cell, Development and Cancer Biology, Knight Cancer Institute, Oregon Health and Science University, Portland, OR USA; 8 Department of Obstetrics and Gynecology, School for Oncology and Developmental Biology, Maastricht University Medical Centre, Maastricht, The Netherlands

**Keywords:** Metastasis, Endometrial cancer

## Abstract

**Background:**

In endometrioid endometrial cancer (EEC), current clinical algorithms do not accurately predict patients with lymph node metastasis (LNM), leading to both under- and over-treatment. We aimed to develop models that integrate protein data with clinical information to identify patients requiring more aggressive surgery, including lymphadenectomy.

**Methods:**

Protein expression profiles were generated for 399 patients using reverse-phase protein array. Three generalised linear models were built on proteins and clinical information (model 1), also with magnetic resonance imaging included (model 2), and on proteins only (model 3), using a training set, and tested in independent sets. Gene expression data from the tumours were used for confirmatory testing.

**Results:**

LNM was predicted with area under the curve 0.72–0.89 and cyclin D1; fibronectin and grade were identified as important markers. High levels of fibronectin and cyclin D1 were associated with poor survival (*p* = 0.018), and with markers of tumour aggressiveness. Upregulation of both *FN1* and *CCND1* messenger RNA was related to cancer invasion and mesenchymal phenotype.

**Conclusions:**

We demonstrate that data-driven prediction models, adding protein markers to clinical information, have potential to significantly improve preoperative identification of patients with LNM in EEC.

## Background

Endometrial cancer (EC) is the most common malignancy of the female reproductive system and the incidence is rising, with 10,677 new cases in the United Kingdom in 2018.^1^ The endometrioid endometrial cancer (EEC) subtype accounts for about 80% of ECs and various subtypes collectively referred to as non-EEC comprise the remaining 20%. The majority of EC patients are diagnosed with early disease and the main treatment is therefore surgical, with hysterectomy and bilateral removal of the ovaries. Although the overall prognosis of EC patients is generally good, with an 80% overall survival at 5 years, still 15–20% of patients with a low-risk profile experience recurrence.^2^ Unfortunately, outcomes for EEC patients with systemic recurrence are horrible, with a median survival hardly exceeding 12 months.^3^

There is little debate whether lymphadenectomy should be performed in patients with non-endometrioid histology or deeply infiltrating high-grade disease, both known to run a more aggressive disease course. However, this is more controversial within the EEC patient group where the risk of lymph node metastasis (LNM) is much lower (8–15%).^4,5^ Although the procedure allows for complete surgical staging and facilitates adjuvant treatment selection, it gives a 10–20% risk of lower-extremity lymphedema and 10–25% risk of lymphocele development.^5–12^ Furthermore, large prospective trials have shown no survival benefit of the procedure.^4,13^ Currently, the indication for lymphadenectomy is based on a clinical risk assessment, including information from tumour histology, and on putative likelihood of LNM.^14,15^ Clinical practice of lymphadenectomy is variable between different countries due to the lack of internationally established criteria.^16^ In Norway, preoperative imaging is part of the clinical risk evaluation, and lymph node dissection is advised in deeply infiltrating grade 3 EEC, as well as in lymph node sampling in grade 1 and 2 deeply infiltrating, and grade 3 superficially infiltrating EEC, as well as in all non-EEC.^17^ However, only a smaller subset of these patients will have LNM confirmed.^18^ As a consequence, many patients will potentially suffer unnecessary complications. The challenge is to better identify the subset of EEC patients with otherwise low-risk profile who have risk of LNMs at presentation;^18^ only these patients should be selected for lymphadenectomy.

Various invasive and non-invasive methods have been suggested for better prediction of LNM in EC patients, including preoperative imaging by different modalities (e.g. magnetic resonance imaging (MRI) and PET/CT) (reviewed in ref. ^19^) sentinel node mapping^20^ and molecular markers.^21^ The ability of preoperative imaging to detect metastases is dependent on lymph node size.^22^ Sentinel node mapping is an invasive procedure, requiring high surgical expertise and may thus not be performed in all hospitals. Also, the efficacy of the procedure is dependent on the accuracy of the dye to correctly map to the sentinel node.^23,24^ In cases where the sentinel node cannot be identified, a full lymph node dissection is warranted.

Consistent alterations of protein levels during tumorigenesis and the metastatic process can be utilised to predict patient outcome. Reverse-phase protein array (RPPA) is an antibody-based dot-blot technology that measures a large number of functional protein levels in a high-throughput manner. Integrating protein markers with clinically available variables (e.g., FIGO (International Federation of Gynaecologist and Obstetrics), stage, grade, age, menopausal status and imaging variables), has the potential to help increase the value of such protein markers.

In this study, our aim was to combine proteins and clinical information to develop robust models that enables identification of EEC patients at high risk of LNM, a patient group that would benefit from more aggressive surgery.

## Methods

### Training and test cohorts

A prospectively collected, population-based EC series with extensive clinical annotation and follow-up data was collected at Haukeland University Hospital, Bergen, Norway from 2001 (REK vest, IRB 2014/1907). Informed written consent was obtained by all included patients and the study was approved by the local ethics committee (REK vest, IRB 2009/2315). Fresh frozen tumour tissue from the operative specimen was available from all cases. Lymph node dissection was performed in 79.3% of patients; only cases where lymphadenectomy was performed were included in our analyses. From this series, we selected two non-overlapping cohorts as training (inclusive of the Bergen training set 2001–2013, *n* = 243) and test set (inclusive of the Bergen test set 2011–2015, *n* = 56), all fully staged and of endometrioid subtype only. Although these cohorts have been manually selected, there were no statistical differences when compared to the whole population-based series (*n* = 1009) on the most important variables (see Supplementary Table 1).

### MDACC cohort

For validation of the low-risk model (low risk in this study is defined as grade 1 and 2 EEC tumours), a cohort from MD Anderson Cancer Centre (MDACC) (Houston, TX, USA; *n* = 100 low-risk EEC patients, Supplementary Table 2) fresh frozen tumour samples along with clinical annotation and follow-up data was used as an external test set. Written consent was signed by all included patients and the study was approved by the local ethics committee (Institutional Review Board of MDACC (Lab08-0580).

### RPPA data analysis

Protein expression data was generated by the RPPA method as previously described.^25–28^ Briefly, proteins were extracted from fresh frozen tumour samples, denatured by sodium dodecyl sulfate and the lysate was 5-fold serially diluted prior to printing on nitrocellulose-coated slides. Proteins were then probed with antibodies that target proteins (see Supplementary Table 3 for antibody information) that participate in major signalling pathways of relevance to cancer.^25,29,30^ The signal was captured using a DakoCytomation-catalysed system and DAB colorimetric reaction. Following antibody probing, RPPA slides were scanned with a customised scanner (Huron Inc.) and spot intensities were analysed and quantified using the Array-Pro Analyser (Meyer Instruments Inc.). Protein expression levels were calculated using the software SuperCurve^31^ (available at http://bioinformatics.mdanderson.org/Software/). Only antibodies with quality control scores >0.8^32^ were included. Heat maps with two-way unsupervised hierarchical clustering analysis were drawn to visualise protein expression patterns. Ward linkage was used as the agglomeration rule and Pearson’s correlation coefficients were used as the dissimilarity metric in hierarchical clustering analysis. Of the profiled proteins, a total of 176 were common between the Bergen training and test sets and 163 proteins overlapped between the Bergen training and MDACC test sets; these were used for all downstream analyses.

### Model development

**Fig. 1:**
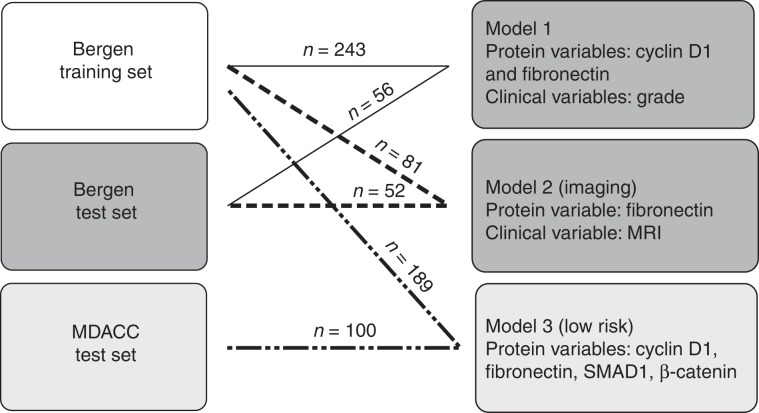
Overview of training and test sets and corresponding models. Lines with number of patients for each set connects the models to the training and test set used in each model. All models were trained using Bergen training (white colour) and tested in either the Bergen test set (dark grey) or in the MDACC test set (light grey).

Three different models were developed (Fig. 1). Model 1 was built on protein variables (*n* = 176) and clinical variables (*n* = 3; age, menopausal status and histologic grade) using the Bergen EEC cohorts as training and test sets. Model 2 comprised of an MRI variable (tumour volume in millilitres) in addition to proteins and clinical variables for *n* = 81 and *n* = 52 of the patients in the Bergen training and test sets, respectively. Model 3 was trained on low-risk (histologic grade 1 and 2) EEC cases from the Bergen cohort and tested in the low-risk MDACC EEC cohort; only protein markers (*n* = 163) were available as variables in this model (see Fig. 1 and results for overview of models and corresponding training and test sets). To identify relevant protein and clinical markers from available variables, LIMMA^33,34^ was used to compare protein expression between groups, and false discovery rate (FDR) <0.05 was considered as significant. A generalised linear model (GLM) was chosen to fit the biomarkers and furthermore selected by Akaike information criterion (AIC) such that only the most informative variables remained. The fitted GLM was used as a logit to a logistic function, which uses the numeric outcomes of the GLM to calculate probabilities. The prediction model was then defined by the logistic function,

1 $$\theta = \frac{{e^{(z)}}}{{1 + e^{(z)}}},$$

where *θ* represents the probability of the occurrence of an event, *e* is the base of the natural logarithm (~2.718), and *z* is the logit GLM, denoted as

2 $$z = \beta _0 + \beta _1x_1 + \beta _2x_2 + \cdots + \beta _kx_k.$$

The logit GLM specifies the linear relationship between event (*z)* and biomarkers (*x*). *β*_0_ is the intercept and *β*_1_, *β*_2_, …, *β*_*k*_ are the regression coefficients of the biomarkers.

### Validation of models

A Binary logistic regression model was used to validate the predictive power of the variables defined by the model; odds ratio (OR) of LNM were evaluated.

### Gene expression microarrays

Gene expression data were available for 203 EEC patients for messenger RNA (mRNA) validation of model 1 and for exploration of gene functions. Microarray analysis was performed as previously described.^35^ In significance analysis of microarrays (SAMs), we dichotomised the mRNA levels for *FN1* and *CCND1*; upper quartile (upper 25%) as high and the lower three quartiles (lower 75%) as low. Two expression profile groups were then annotated; the *high* expression group was defined as both *FN1* and *CCND1* highly expressed (*n* = 26) and *low/discordant* expression group was defined as all other combinations (i.e. high and low, low and high, low and low) (*n* = 177). A fold change of 1.2 and FDR <0.1% were used as cut-off and analyses were performed using the J-Express software (Molmine, Bergen, Norway). Molecular signatures database (MSigDB, version 5.1) was used to compute overlap of differentially expressed transcripts with Gene Ontology gene sets (C5) and curated gene sets (C1).

### Statistical analyses for survival and association to clinical data

Statistical analyses were performed using the statistical program SPSS-25 (IBM, New York). Pearson’s *χ*^2^ test was used to evaluate the associations between categorical variables. The differential distribution of continuous variables between two categorical variables was assessed by *t* test or Mann–Whitney *U* test for normal and non-normal distributed data, respectively. Correlation of continuous variables was assessed by Pearson’s correlation coefficient. Survival differences between the groups were assessed by Kaplan–Meier using the Mantel–Cox (log-rank) test. Univariate survival analyses were performed for disease-specific survival (DSS), with death due to EC defined as an event. Patient’s death from other causes was censored at the time of death.

## Results

### Patient characteristics

Comparing clinicopathological characteristics of the Bergen training and Bergen test sets, a higher percentage of pre-/perimenopausal patients was noted in the training cohort: 14.0% compared to 3.6% in the test set (*χ*^2^, *P* = 0.03, Table 1). In the test set, a higher proportion of patients had paraaortic in addition to pelvic lymph nodes removed (*χ*^2^ test, *P* < 0.001) due to change in surgical routines in more recent years. Overall, there were no significant differences in FIGO 2009 stage between the training and test cohorts (*χ*^2^ test, *P* = 0.73). Further, no significant differences were identified between training and test set for age, histologic grade, lymphadenectomy, recurrences and disease-specific deaths (*χ*^2^ test or Mann–Whitney *U* test, *P* = 0.21, 0.65, 0.93, 0.14, and 0.31).

**Table 1:** Clinical characteristics of the Bergen training and test cohort.

Variable	Training set	Test set	*P* value^a^
	*n* (%)	*n* (%)	
Inclusion time	2001–2013	2011–2015	
Number of patients	243	56	
Age (median, range)	63 (32–89)	65 (38–88)	0.20
Figo stage			0.73
I	191 (78.6)	44 (78.6)	
II	20 (8.2)	4 (7.1)	
III	28 (11.5)	8 (14.3)	
IV	4 (1.6)	0 (0.0)	
Histologic grade^b^			0.65
Grades 1 and 2 (low risk)	189 (79.1)	45 (81.8)	
Grade 3 (high risk)	50 (20.9)	10 (18.2)	
Menopausal status			**0.03**
Pre/perimenopausal	34 (14.0)	2 (3.6)	
Postmenopausal	209 (86.0)	54 (96.4)	
Lymphadenectomy			0.93
Positive nodes	27 (11.1)	6 (10.7)	
Negative nodes	216 (88.9)	50 (89.3)	
Number of nodes removed (median, range)^c^	13 (0–49)	15 (1–35)	0.11
Type of nodes removed			**<0.001**
Pelvic	237 (97.5)	47 (83.9)	
Pelvic and paraaortic	6 (2.5)	9 (16.1)	
Recurrences	46 (19.7)	6 (11.1)	0.14
Death due to disease	34 (14.0)	5 (8.9)	0.31
Follow-up time (median, range)	64 (0–168)	45 (1–58)	**<0.001**

*FIGO* International Federation of Gynaecologist and Obstetrics, *SD* standard deviation, *n* number of patients.

*P*-values marked in bold indicate numbers that are significant on the 95% confidence limit.

^a^Categorical variables: Pearson’s *χ*^2^ test. Continous variables: Mann–Whitney *U* test.

^b^Missing information for four patients in the Norwegian Training Cohort and for one patient in the Norwegian Test Cohort.

^c^Missing information for one patient in the Norwegian Training Cohort.

The Bergen training cohort and the MDACC test cohort, used together in model 3, differed significantly for age, FIGO stage, histologic grade and body mass index (BMI) (continuous and categorical) (all *P* < 0.05, Supplementary Table 4). Although borderline non-significant (*χ*^2^ test, *P* = 0.054), a higher percentage of patients with metastatic lymph nodes were identified in the MDACC cohort (i.e. 16% MDACC vs. 8.5% Bergen cohort).

### Model 1 predicts LNM in EEC

**Fig. 2:**
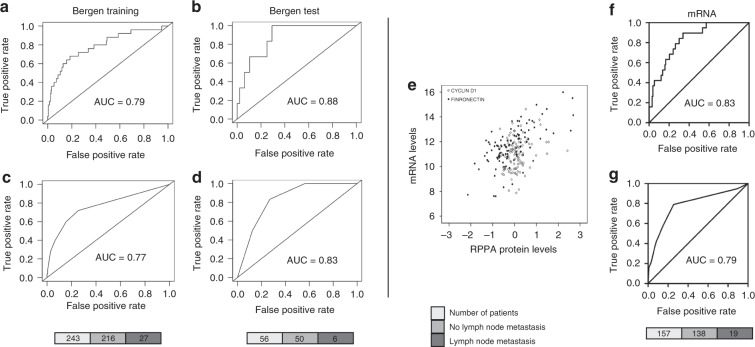
Prediction model (model 1) and RNA confirmation. Receiver operating characteristic (ROC) curves for model 1 used as a continuous model (**a**, **c**) and as a categorical model (**b**, **d**) in the Bergen training and test cohorts. The number of patients is shown in the lower panel (grey scale). **e** Scatter plot of cyclin D1 and fibronectin RPPA protein levels vs. mRNA levels. Black round dots and square white dots illustrate fibronectin/*FN1* and cyclin D1/*CCND1* expression levels for each case, respectively. RNA validation for model 1 used as a continuous model (**f**), and as a categorical model (**g**). The number of patients is shown in the lower panel.

To identify differentially expressed proteins between lymph node-positive and lymph node-negative groups, we first performed a LIMMA analysis from completely staged patients (defined as at least pelvic lymphadenectomy performed). LIMMA analysis revealed that 24 proteins were significantly altered between these groups (FDR <0.05). Of most interest, fibronectin, cyclin D1 and Stathmin were upregulated and E-cadherin, B-cell lymphoma 2 (Bcl-2) and oestrogen receptor (ER) were downregulated in lymph node-positive cases (Supplementary Table 4). Significantly different proteins were then run together with three clinical variables in a GLM, which was further subjected to stepwise selection by (AIC) such that only the most informative variables remained. The model was defined by the following logistic function: (−0.1000) + 0.2012 × grade + 0.1631 × cyclin D1 + 0.0699 × fibronectin, where cyclin D1 and fibronectin are the relative protein levels by RPPA and grade is a dichotomisation with ‘low-risk’ (grade 1 or 2 EECs) and ‘high-risk’ (grade 3 EECs). The model predicted patients with LNM with a area under the curve (AUC) of 0.79 and 0.88 for the Bergen training and test set, respectively (Fig. 2a, b). The model remained highly significant (*P* < 0.001) also when validated using a binary logistic regression model, as described in Methods. Additionally, binary logistic regression analysis of the individual variables confirmed the importance of combining all three variables in the prediction algorithm (Supplementary Fig. 1).

To test the robustness of the model for a clinical setting where tests are often dichotomised, we ran the model again using a protein cut-off for *high* expression (upper 25%) and *low* expression (lower 75%). The resulting categorical model predicted LNM with an AUC of 0.77, which was validated in the test set with an AUC of 0.83 (Fig. 2c, d). To potentially validate our findings using mRNA expression data (i.e. *FN1* and *CCND1*), protein expression was correlated to mRNA expression levels. *FN1* and *CCND1* mRNA significantly correlated to fibronectin and cyclin D1 protein levels, respectively (fibronectin: Spearman’s correlation analysis, *r* = 0.619, *P* < 0.001; cyclin D1: Pearson’s correlation analysis, *r* = 0.227, *P* = 0.020) (Fig. 2e). mRNA expression data and histologic grade were included in a binary logistic regression analysis. The model predicted LNMs with an AUC of 0.83 in the continuous model and 0.79 in the categorical model (Fig. 2f, g). Of note, *CCND1* mRNA was not significant (*P* = 0.106) and was thus excluded from the analysis (the model thus consisted of histologic grade and *FN1* mRNA).

### Model 2 adds potential value to the prediction

**Fig. 3:**
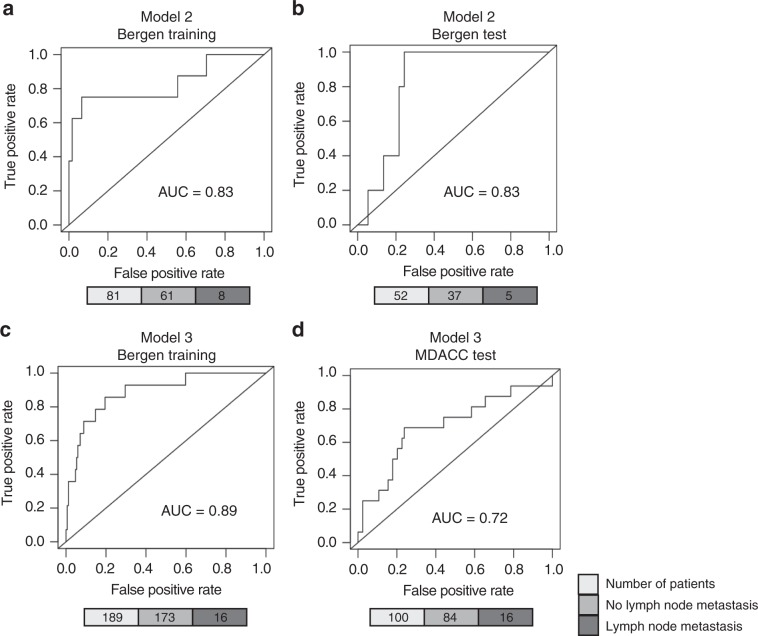
Alternative lymph node metastasis prediction models using MRI and protein data (model 2) or protein data only (model 3). Receiver operating characteristic (ROC) curves for model 2, using MRI and protein data in the Bergen training (**a**) and the Bergen test cohort (**b**), and model 3, using significantly altered proteins only in a subpopulation of presumed low-risk patients in the Norwegian training cohort (**c**) and the MDACC test cohort (**d**). The number of patients is shown in the lower panel (grey scale).

In a number of countries including Norway, MRI is part of the clinical work-up of patients with (suspicion of) EC. In model 2, MRI was thus added to the clinical variables to test if it can improve the performance of the model and mimic the clinical situation in those companies. Preoperative MRI was performed routinely for all patients included after 2011 to assess tumour size, invasion depth and suspicion of metastasis. In the Bergen training cohort, LIMMA analysis now identified five proteins as significantly differentially expressed (all FDR <0.05) between cases with and without LNM. Again, following LIMMA analysis, the significantly different proteins were run in a GLM that selects only the most informative proteins; in this case, only one protein remained. A prediction model was ultimately defined by the following logistic function: 0.0249 + 0.0060 × MRIvol + 0.0866 × fibronectin, where MRIvol is tumour volume in millilitres and fibronectin is the relative protein level measured by RPPA. LNM was predicted with an AUC of 0.83 in both training and test sets (Fig. 3a, b). These findings were confirmed by binary logistic regression modelling (*P* < 0.001). Although fibronectin appears to be a stronger predictor in individual modelling, both variables contributed to the results (Supplementary Fig. 2).

### Model 3 predicts LNM in low-risk EEC patients

Model 3 is a protein-only model developed in the lowest-risk EEC patients; those with histologic grades 1 and 2, thus presumed the lowest risk of LNM. However, a small subset of these patients (up to 8%) will have metastatic nodes at presentation and should be identified. To explore how accurate our model could predict LNM in this lowest-risk group, we used the Bergen training cohort as the training set (*n* = 189 low-risk cases). LIMMA analysis identified eight proteins as significantly differentially expressed between the groups with positive and negative lymph node status (all FDR <0.05). A prediction model was defined by the following logistic function: 0.0997 + 0.1741 × cyclin D1 – 0.2083 × SMAD1 + 0.0466 × fibronectin – 0.0374 × β-catenin, where all variables are relative protein levels measured by RPPA. The model predicted patients at risk of nodal metastasis with an AUC of 0.89 in the Bergen training set (Fig. 3c). Despite significant differences in training and test set characteristics, the prediction was validated in the MDACC test set with an AUC of 0.72 (Fig. 3d).

### Fibronectin and cyclin D1 RPPA protein levels correlate with aggressive characteristics

As fibronectin and cyclin D1 were identified as key proteins for the prediction models, we examined the individual proteins in relation to important clinicopathological factors (Table 2). A high level (see model 1 for definition of high and low level) of fibronectin was, in addition to a positive association with metastatic lymph nodes (*P* < 0.0001), associated with high FIGO stage (*P* = 0.001), high histologic grade (*P* = 0.001) and preoperative risk classification (*P* = 0.03). High levels of cyclin D1, correlated significantly to LNM (*P* < 0.001), as expected, and additionally with FIGO stage (*P* < 0.001), deep myometrial infiltration (*P* = 0.002) and menopausal status (*P* = 0.04).

**Table 2:** Fibronectin and cyclin D1 expression in relation to clinicopathological factors in the Bergen training set.

Variable	Fibronectin (RPPA)		*P* value	Cyclin D1 (RPPA)		*P* value
	High *n* (%)	Low *n* (%)		High *n* (%)	Low *n* (%)	
Number of patients	64 (26.3)	179 (73.7)		64 (26.3)	179 (73.7)	
Age, median			0.86			0.87
<66 years	38 (26.8)	104 (73.2)		35 (24.6)	107 (75.4)	
≥66 years	26 (25.7)	75 (74.3)		24 (23.8)	77 (76.2)	
Figo stage			**0.001**			**<0.001**
I	40 (20.9)	151 (79.1)		37 (19.4)	154 (80.6)	
II	7 (35.0)	13 (65.0)		3 (15.0)	17 (85.0)	
III	16 (57.1)	12 (42.9)		16 (57.1)	12 (42.9)	
IV	1 (25.0)	3 (75.0)		3 (75.0)	1 (25.0)	
Menopausal status			0.20			**0.04**
Pre/perimenopausal	12 (35.3)	22 (64.7)		13 (38.2)	21 (61.8)	
Postmenopausal	52 (24.9)	157 (76.1)		46 (22.0)	163 (78.0)	
Histologic grade^a^			**0.001**			0.33
Grades 1 and 2	41 (21.7)	148 (78.3)		44 (23.3)	145 (76.7)	
Grade 3	22 (44.0)	28 (56.0)		15 (30.0)	35 (70.0)	
Preoperative risk classification^b^			**0.03**			0.28
Low	54 (24.3)	168 (75.7)		51 (23.0)	171 (77.0)	
High	7 (50.0)	7 (50.0)		5 (35.7)	9 (64.3)	
Recurrence^c^			0.20			0.06
No	44 (23.4)	144 (76.6)		37 (19.7)	151 (80.3)	
Yes	15 (32.6)	31 (67.4)		15 (32.6)	31 (67.4)	
Lymphadenectomy			**<0.001**			**<0.001**
Negative nodes	48 (22.2)	168 (77.8)		42 (19.4)	174 (80.6)	
Positive nodes	16 (59.3)	11 (40.7)		17 (63.0)	10 (37.0)	
Myometrial infiltration			0.50			**0.002**
<50%	33 (24.6)	101 (75.4)		22 (16.4)	112 (83.6)	
≥50%	31 (28.4)	78 (71.6)		37 (33.9)	72 (66.1)	

*FIGO* International Federation of Gynaecologist and Obstretics, *n* number of patients.

*P*-values marked in bold indicate numbers that are significant on the 95% confidence limit.

^a^Data missing for four patients.

^b^Data missing for seven patients.

^c^Data missing for nine patients.

**Fig. 4:**
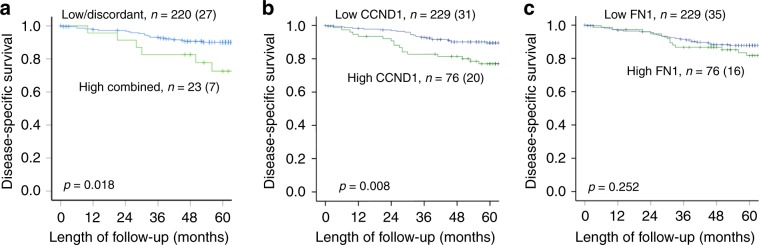
Disease-specific survival in patients with high vs. low fibronectin and cyclin D1 levels. Disease-specific survival (DSS) according to fibronectin and cyclin D1 high combined (i.e. 25% upper quartile of both proteins) vs. low/discordant expression (i.e. all other combinations: 75% lower quartile of at least one protein) (**a**). DSS was also calculated for the individual variables: fibronectin (**b**) and cyclin D1 (**c**) high (upper 25% quartile) and low (lower 75% quartile) expression. The number of events is given within parentheses.

As both aggressive characteristics and LNM increase risk of poor survival, differences in survival between high and low expression groups were explored for fibronectin and cyclin D1. Combined high expression of both fibronectin and cyclin D1 (both upper 25% quartiles) was associated with poor prognosis compared to patients with a discordant or low expression (*P* = 0.018) (Fig. 4a). We also explored survival differences for each protein alone; interestingly, high cyclin D1 expression was associated with poorer survival (*P* = 0.008) and separated the groups even better than the proteins combined (Fig. 4b). Fibronectin expression levels did not show a significant association with survival (*P* = 0.252) (Fig. 4c).

### Fibronectin- and cyclin D1-related genes are linked to an invasive tumour phenotype

We further explored the combined role of *FN1* and *CCND1* in EEC in a microarray mRNA expression dataset. A SAM was performed between high and low mRNA expression groups (see Method section for a definition of the expression groups) and identified 361 transcripts to be significantly differently expressed (FDR <0.001 and fold change >1.2 was used as cut-off) and, of these, 199 transcripts were detected as significantly upregulated in tumours of *high FN1* and *CCND1* expression (see Supplementary Table 5 for the list of top 40 differentially expressed genes). Submitting our gene list to the MSigDB identified gene sets characteristic of an invasive phenotype in tumours of *high FN1*/*CCND1* expression (Supplementary Table 6), including upregulation of gene sets related to extracellular matrix, cell movement, cancer-specific invasion and mesenchymal phenotype. In contrast, gene sets associated with *low or discordant FN1*/*CCND1* expression profile were related to a less aggressive phenotype, that is, cilia morphology and non-invasive characteristics.

## Discussion

It is challenging to preoperatively accurately identify the subset of EEC patients with metastatic lymph nodes and thus advanced disease, who, at the time of hysterectomy, would benefit from a lymphadenectomy procedure and subsequently adjuvant treatment. Due to the substantial risk of procedure-related complications, lymphadenectomy is not performed in some patients who ideally should have had the procedure performed and also vice versa. The latter thus introduces an unnecessary risk of lower-extremity lymphedema and lymphocele development. More knowledge is specifically needed in the endometrioid subtype as this is the most common in EC and the a priori risk of LNM is relatively low in this disease subset (8–15%).^4,5^ Our aim was therefore to develop an integrative model using readily available clinical and protein variables that together provide a better identification of EEC patients with LNM.

Ultimately three models were developed, varying the available input variables and patient selection. The main model (model 1), including fibronectin and cyclin D1 combined with preoperatively available tumour grade, is to our knowledge the first protein-driven model to predict LNM in EEC. MRI has long been established as a valuable preoperative imaging tool in EC and was added in model 2. Its capability to contribute to preoperative prediction of LNM has been demonstrated by us and others.^36,37^ When tumour size on MRI was added to the variables (model 2), only one protein remained in the model with AUC values similar to the original model, suggesting that MRI significantly improved the prediction accuracy of the model. Due to the lower number of patients with MRI available, model 2 should be validated in larger cohorts to increase statistical power. Integrative approaches, incorporating routinely used clinical tools such as imaging in prediction models, can facilitate their translation to and applicability in clinical practice. In future studies, adding a combination of quantitative imaging features, easily extracted from MR images, might further increase the model performance and has shown promise for prediction of prognosis and LNM in several cancers.^38–42^

The robustness of our approach was further reflected in model 3 (low-risk model), which only includes patients who according to most guidelines are no longer advised lymphadenectomy (grade 1 and 2 EEC), overlooking the small unidentified subset that has metastatic nodes at diagnosis. Thus, especially here, patients could benefit from risk models to ensure that those grade 1 and 2 patients, who do have high risk of nodal metastasis, are offered lymph node dissection. Even in this lowest-risk cohort, the performance of the model is encouraging with an AUC of 0.82 (training) and 0.72 (test set), suggesting that use of the model may be instrumental to better identify high-risk patients in this subset. The significant intrinsic differences between the MDACC cohort and the Bergen cohort are a likely explanation for the lower AUC in the test set. Unfortunately, the available preoperative clinical variables were not identical between the sets in model 3 and thus only protein data could be tested here. It will be interesting to test in subsequent studies whether this model can be further optimised by integration of clinical variables. The challenge may lie in the fact that lymph node dissection, as mentioned, often is omitted in this patient population and that thus further retrospective datasets to confirm the findings may be sparse. Finally, it should be noted that the lowest-risk cohorts only included patients with confirmed lymph node status who all underwent lymphadenectomy. Thus, a subpopulation of very low-risk EEC patients has not been included in our analysis, potentially introducing a selection bias in our dataset.

For a model to be robust and more easily transferable to the clinic, ideally a low number of variables is needed. Our main model and its two variations demonstrate that a high predictability can be combined with a low number of variables (proteins and clinical variables). Additionally, it showed good performance on dichotomisation, again facilitating clinical applicability.

For clinical implementation of the model, it would be interesting to validate our findings using immunohistochemistry (IHC). We tested two antibodies for this purpose: anti-fibronectin (FBN11, MA5-11981, Thermo Fisher Scientific) and anti-cyclin D1 (SP4, MA5-16356, Thermo Fisher Scientific). Unfortunately, neither of these correlated well enough to RPPA protein levels to be included in the model (data not shown). A significant challenge was that the antibodies from the RPPA platform were unavailable for IHC. Further testing of IHC-supported antibodies is thus needed to validate whether RPPA protein levels can be replaced by IHC staining of patient tissue in our LNM prediction model. It is however interesting to note that mRNA levels of *FN1* and *CCND1* corresponded well with RPPA levels, suggesting that quantitative PCR might be an alternative method for testing. Further, other approaches such as Nanostring with bar-coded antibodies may also allow translation to the clinic as we have done with other targets.^43^

The strong association of fibronectin and cyclin D1 with characteristics of tumour aggressiveness such as higher FIGO stage and grade and poorer DSS suggests their clinical relevance. This is supported by literature showing fibronectin to be a well-established marker of the epithelial–mesenchymal transition (EMT).^44^ Also, functional studies have linked fibronectin to EMT and cell migratory behaviour.^45,46^ Upregulated fibronectin activates a set of signalling pathways, such as EGFR and HER2 signalling, which in turn are a feedback to stimulate fibronectin expression.^45,47,48^ Although fibronectin has previously been linked to LNM in breast and oral cancer, this is the first time the protein has been associated with metastasis in EC.

Cyclin D1 is a pleiotropic protein that is best known as a cell cycle protein, but it has also been shown to facilitate cell mobility and cytoskeletal remodelling,^49,50^ and associated with increased metastasis.^49^ The significance of increased expression of cyclin D1 is consistent with findings by Du et al.^51^ who developed an RPPA protein-based prediction model to help distinguish early- from late-stage EEC patients. Contrasting our own model, their model was not specific to the prediction of LNM, which may explain the differences in proteins and pathways represented, such as HER3, SHC and JNK. However, the increased expression of the Wnt-pathway protein Dvl3 is in line with our findings as Dvl3 induces cyclin D1 expression, thus activating the same pathway. Consistently, Wnt activation has been shown as a driver of lung cancer metastasis,^52,53^ and has also been associated with metastasis in EC.^54^ In addition to cyclin D1 and fibronectin, our LIMMA analysis identified an upregulation of Stathmin and a downregulation of E-cadherin, Bcl-2 and ER. Similar alterations in expression patterns of the individual proteins have previously been demonstrated and extensively linked to LNM by us and others.^18,55–57^ These findings support that LIMMA correctly identifies proteins associated with nodal metastasis and indicate that multiple proteins are involved; our model only selects the most contributing proteins. However, functional studies are needed to build up a picture of protein networks that drives metastasis. It is also important to note that LIMMA analysis is performed on a panel of cancer-related proteins selected for RPPA. Thus, it is likely that additional proteins would be identified with a more global approach.

Yang et al.^58^ developed another RPPA model aimed to improve prognostication in EC by incorporating protein and clinical data into a model for low stage (FIGO stage 1 and 2) and high stage (FIGO stage 3 and 4), with overall survival as main outcome variable. They showed that in early-stage disease, their model outperformed individual clinical variables, including stage and grade. Again, the focus in their study, discriminating patients by prognosis, differed from our aim to identify LNM. Judging from the complexity of the models (ref.,^59^ 14–18 proteins and 2 clinical variables vs. 1–4 proteins and 0–1 clinical variable in this study) it may be hypothesised that the process of LNM is less variable, and dependent on less pathways compared to ‘prognosis’ in general. However, similar to Yang et al.,^58^ we feel integrative models are of potential clinical utility to identify patients with assumed early-stage EEC, who may benefit from more aggressive surgery and who otherwise might not have been identified. Interestingly, their training cohort overlapped to a large extent with our training cohort, thus proving that the different selection of proteins is indeed related to the question posed and not cohort related.

Finally, the strong association of fibronectin and cyclin D1 with LNM was supported by our gene expression analyses, giving further biologic insight. Further functional studies should determine to what degree these proteins are *drivers* of metastasis, which was not within the scope of this study.

Taken together, these findings suggest that the proteins in our models are associated with LNM, especially when co-expressed in EC. Algorithms reliably predicting lymph node involvement are highly valuable, supplementing the current risk stratification: to detect patients with the highest risk of nodal disease, including the lowest-risk patients. An interesting thought may be that if we can accurately identify the patients with LNM preoperatively and the procedure itself, shown by two large RCTs,^4,13^ does not convey prognostic benefit for the patient, the procedure can be omitted altogether and effective adjuvant treatment can be offered to these patients. We have not come so far yet, but through optimisation of models this may become in reach and be beneficial in this often pre-existing (multi)morbid and obese population.

## Supplementary information

Supplementary Figures and Tables

Supplementary Table 2

## Data Availability

N/A
