# Supplementary material for: Development of prediction models for lymph node metastasis in endometrioid endometrial carcinoma
Source: Br J Cancer. 2020 Feb 10;122(7):1014–22. doi: 10.1038/s41416-020-0745-6 (PMC7109044; doi:10.1038/s41416-020-0745-6)
Supplement: Supplementary file 1 — Supplementary Figures and Tables [file 41416_2020_745_MOESM1_ESM.docx]

**Supplementary Tables.**

**Supplementary Table 1. Comparison of Bergen Biobank Prospective cohort and Bergen training cohort.**

|  | **Prospective series** |  | **Bergen training cohort** |  |
| --- | --- | --- | --- | --- |
| Variable | n (%) |  | n (%) | P-value |
| Number of patients | 1009 |  | 243 |  |
| **Age (median, range)** | 64 (25-90) |  | 63 (32-89) | 0.29 |
| **Figo stage** |  |  |  | 0.92 |
| I | 812 (80.5) |  | 191 (78.6) |  |
| II | 79 (7.8) |  | 20 (8.2) |  |
| III | 103 (10.2) |  | 28 (11.5) |  |
| IV | 15 (1.5) |  | 4 (1.6) |  |
| **Histologic grade^a^** |  |  |  | 0.13 |
| Grade 1-2 | 829 (82.2) |  | 189 (77.8) |  |
| Grade 3 | 167 (16.6) |  | 50 (20.6) |  |
| **Lymphadenectomy** |  |  |  | 0.27 |
| Positive nodes | 89 (8.8) |  | 27 (11.1) |  |
| Negative nodes | 920 (91.2) |  | 216 (88.9) |  |

Abbreviations: FIGO: International Federation of Gynecology and Obstretics; n=number of patients.
^a^: data missing for 13 patients in prospective series and for 4 patients in Bergen training cohort.

**Supplementary Table 2. List of antibodies used for RPPA.**

**See supplementary Excel file.**

**Supplementary Table 3. Clinical characteristics of Bergen training and MDACC test cohorts.**

|  | **Training Set** |  | **Test Set** |  |  |
| --- | --- | --- | --- | --- | --- |
| Variable | n (%) |  | n (%) |  | P-value^a^ |
| Inclusion time | 2001-2013 |  | 2000-2009 |  |  |
| Number of patients | 189 |  | 100 |  |  |
| **Age (median, range)** | 63 (32-88) |  | 57 (24-90) |  | **0.01** |
| **Figo stage** |  |  |  |  | **0.03** |
| I | 156 (83.0) |  | 68 (68.0) |  |  |
| II | 14 (7.4) |  | 15 (15.0) |  |  |
| III | 16 (8.5) |  | 14 (14.0) |  |  |
| IV | 2 (1.1) |  | 3 (3.0) |  |  |
| **Histologic grade^b^** |  |  |  |  | **< 0.001** |
| Grade 1 | 89 (47.3) |  | 21 (21.0) |  |  |
| Grade 2 | 99 (52.7) |  | 79 (79.0) |  |  |
| **Lymphadenectomy** |  |  |  |  | 0.05 |
| Positive nodes | 16 (8.5) |  | 16 (16.0) |  |  |
| Negative nodes | 172 (91.5) |  | 84 (84.0) |  |  |
| **BMI (median, range)^c^** | 27 (16-55) |  | 34 (19-74) |  | **< 0.001** |
| **BMI groups^d^** |  |  |  |  | **< 0.001** |
| 20-25 | 52 (32.5) |  | 9 (9.9) |  |  |
| 25-30 | 50 (31.3) |  | 22 (24.2) |  |  |
| 30-35 | 32 (20.0) |  | 18 (19.8) |  |  |
| 35-40 | 15 (9.4) |  | 12 (13.2) |  |  |
| > 40 | 11 (6.9) |  | 30 (33.0) |  |  |

Abbreviations: BMI: Body mass index; FIGO: International Federation of Gynecology and Obstretics; SD: Standard deviation; n=number of patients.

^a^: categorical variables pearson chi-square test, continuous variables Mann-Whitney U test; ^b^: data missing for 3 patients in Norwegian training cohort; ^c^: data missing for one patient in MDACC test cohort; ^d^: data missing for 28 patients in Norwegian training cohort and for 9 patients in MDACC test cohort.

**Supplementary Table 4: Differentially expressed proteins between
patients with and without lymph node metastasis.**

| **Norwegian training cohort (n=243)** | | |
| --- | --- | --- |
| **Protein** | **Log2 fold change (LN metastasis vs no LN metastasis)** | **FDR adjusted p-value** |
| CYCLIND1 | 0.42 | 0.0003 |
| FIBRONECTIN | 0.77 | 0.0008 |
| ECADHERIN | -0.85 | 0.0018 |
| BID | 0.15 | 0.0045 |
| BCATENIN | -0.65 | 0.0211 |
| HSP70 | 0.34 | 0.0297 |
| PKCPANBIIPS660 | -0.33 | 0.0349 |
| STATHMIN | 0.20 | 0.0083 |
| RAD51 | 0.25 | 0.0183 |
| AMPKA | -0.21 | 0.0209 |
| NOTCH1 | 0.14 | 0.0296 |
| BADPS112 | -0.19 | 0.0428 |
| X4EBP1 | 0.47 | 0.0026 |
| FASN | 0.45 | 0.0349 |
| TFRC | 0.53 | 0.0428 |
| BACTIN | 0.30 | 0.0322 |
| MIG6 | 0.11 | 0.0168 |
| STAT3PY705 | -0.31 | 0.0296 |
| PDCD4 | -0.52 | 0.0297 |
| SMAD1 | -0.18 | 0.0083 |
| YAPPS127 | -0.43 | 0.0111 |
| ER | -1.13 | 0.0026 |
| BCL2 | -0.48 | 0.0057 |
| HER3PY1289 | 0.10 | 0.0209 |
|  |  |  |
| **Norwegian test cohort (n=56)** | | |
| **Protein** | **Log2 fold change (LN metastasis vs no LN metastasis)** | **p-value (unadjusted)** |
| P53 | 0.61 | 0.0006 |
| ECADHERIN | -0.96 | 0.0082 |
| HER3 | 0.43 | 0.0101 |
| RAB25 | -0.50 | 0.0216 |
| TTF1 | 0.17 | 0.0258 |
| XRCC1 | 0.32 | 0.0275 |
| GAB2 | 0.37 | 0.0290 |
| CDK1 | 0.57 | 0.0314 |
| PAXILLIN | 0.49 | 0.0322 |
| PI3KP110A | 0.26 | 0.0429 |

**Supplementary Table 5. Differentially expressed genes in tumours with high versus low expression of *FN1* and *CCND1*.**

A. Top 20 upregulated genes in patients with high vs low *FN1* and *CCND1*.

| **GeneName** | **Description** | **FDR** | **Fold change** |
| --- | --- | --- | --- |
| FN1 | Fibronectin 1 [NM_054034] | < 0.001 | 5.39 |
| SFRP2 | Secreted frizzled-related protein 2 [NM_003013] | < 0.001 | 4.91 |
| MMP1 | Matrix metallopeptidase 1 [NM_002421] | < 0.001 | 3.46 |
| AREG | Amphiregulin [NM_001657] | < 0.001 | 3.22 |
| CTHRC1 | Collagen triple helix repeat containing [NM_138455] | < 0.001 | 3.10 |
| THBS2 | Thrombospondin 2 [NM_003247] | < 0.001 | 3.02 |
| CCND1 | Cyclin D1[NM_053056] | < 0.001 | 2.88 |
| AL359062 | mRNA full length insert cDNA clone EUROIMAGE 1913076 [AL359062] | < 0.001 | 2.81 |
| INHBA | Inhibin. beta A [NM_002192] | < 0.001 | 2.79 |
| SLC6A14 | Solute carrier family 6 [NM_007231] | < 0.001 | 2.75 |
| NNMT | Nicotinamide N-methyltransferase [NM_006169] | < 0.001 | 2.74 |
| S100A3 | S100 calcium binding protein A3 [NM_002960] | < 0.001 | 2.71 |
| PHLDA1 | Pleckstrin homology-like domain, family A, member 1 [NM_007350] | < 0.001 | 2.70 |
| AK001903 | cDNA FLJ11041 fis. clone PLACE1004405 [AK001903] | < 0.001 | 2.66 |
| GPC6 | Glypican-6 precursor [ENST00000377047] | < 0.001 | 2.65 |
| COL11A1 | Collagen, type XI, alpha 1 [NM_080629] | < 0.001 | 2.64 |
| TMEM45A | Transmembrane protein 45A [NM_018004] | < 0.001 | 2.60 |
| CYP24A1 | Cytochrome P450, family 24, subfamily A, polypeptide 1 [NM_000782] | < 0.001 | 2.53 |
| POSTN | Periostin, osteoblast specific factor [NM_006475] | < 0.001 | 2.52 |
| FBN1 | Fibrillin 1 [NM_000138] | < 0.001 | 2.48 |

B. Top 20 downregulated genes in patients with high *FN1* and *CCND1* vs low.

| **GeneName** | **Description** | **FDR** | **Fold change** |
| --- | --- | --- | --- |
| C10orf79 | Chromosome 10 open reading frame 79 [NM_025145] | < 0.001 | -3.64 |
| TSGA2 | Testis specific A2 homolog [NM_080860] | < 0.001 | -3.49 |
| TTC18 | Tetratricopeptide repeat domain 18 [NM_145170] | < 0.001 | -3.14 |
| HOXB5 | Homeobox B5 [NM_002147] | < 0.001 | -3.09 |
| C1orf88 | Chromosome 1 open reading frame 88 [NM_181643] | < 0.001 | -3.04 |
| TTC25 | Tetratricopeptide repeat domain 25 [NM_031421] | < 0.001 | -3.03 |
| SPAG8 | Sperm associated antigen 8 [NM_172312] | < 0.001 | -2.95 |
| FOXN4 | Forkhead box N4 [NM_213596] | < 0.001 | -2.88 |
| TEKT2 | Tektin 2 [NM_014466] | < 0.001 | -2.81 |
| RNF183 | Ring finger protein 183 [NM_145051] | < 0.001 | -2.80 |
| LOC129881 | Hypothetical LOC129881 [BC117445] | < 0.001 | -2.71 |
| AF289562 | Clone pp6337 [AF289562] | < 0.001 | -2.69 |
| UNG2 | Uracil-DNA glycosylase 2 [NM_021147] | < 0.001 | -2.68 |
| LOC339778 | cDNA FLJ34164 fis, clone FCBBF3014567. [AK091483] | < 0.001 | -2.64 |
| LRRC48 | Leucine rich repeat containing 48 [NM_031294] | < 0.001 | -2.38 |
| CASC1 | Cancer susceptibility candidate 1 [NM_018272] | < 0.001 | -2.30 |
| TTC21A | Tetratricopeptide repeat domain 21A [NM_145755] | < 0.001 | -2.24 |
| KIAA1407 | KIAA1407 [NM_020817] | < 0.001 | -2.17 |
| RIBC1 | RIB43A domain with coiled-coils 1 [NM_144968] | < 0.001 | -2.15 |
| LOC90835 | Hypothetical protein LOC90835 (LOC90835) [NM_001014979] | < 0.001 | -2.00 |

**Supplementary Table 6: Gene set enrichment analysis (GSEA) comparing high vs low *FN1* and *CCND1* expression in EEC patients using C5 (GO) gene sets and C2 (curated) gene sets.**

A. Selected upregulated gene sets from top 50 ranked signalling pathways

| **Rank** | **Gene Set Name** | **Genes in  Overlap** | **FDR (%)** | |
| --- | --- | --- | --- | --- |
|  | **C5: *GO gene sets*** |  |  |  |
| 1 | GO_BIOLOGICAL_ADHESION | 44 | < 0.001 |  |
| 2 | GO_LOCOMOTION | 45 | < 0.001 |  |
| 3 | GO_EXTRACELLULAR_MATRIX | 28 | < 0.001 |  |
| 4 | GO_EXTRACELLULAR_STRUCTURE_ORGANIZATION | 25 | < 0.001 |  |
| 6 | GO_COLLAGEN_CONTAINING_EXTRACELLULAR_MATRIX | 24 | < 0.001 |  |
| 7 | GO_CELL_MOTILITY | 40 | < 0.001 |  |
| 11 | GO_SKELETAL_SYSTEM_DEVELOPMENT | 22 | < 0.001 |  |
| 13 | GO_REGULATION_OF_CELL_POPULATION_PROLIFERATION | 34 | < 0.001 |  |
| 27 | GO_CONNECTIVE_TISSUE_DEVELOPMENT | 15 | < 0.001 |  |
| 33 | GO_POSITIVE_REGULATION_OF_LOCOMOTION | 19 | < 0.001 |  |
| 34 | GO_CIRCULATORY_SYSTEM_DEVELOPMENT | 25 | < 0.001 |  |
| 35 | GO_POSITIVE_REGULATION_OF_CELL_POPULATION_PROLIFERATION | 23 | < 0.001 |  |
| 40 | GO_INTEGRIN_BINDING | 11 | < 0.001 |  |
| 48 | GO_BLOOD_VESSEL_MORPHOGENESIS | 19 | < 0.001 |  |
|  | **C2: *Curated gene sets*** |  |  |  |
| 1 | SCHUETZ_BREAST_CANCER_DUCTAL_INVASIVE_UP | 36 | < 0.001 |  |
| 2 | NABA_MATRISOME | 42 | < 0.001 |  |
| 3 | POOLA_INVASIVE_BREAST_CANCER_UP | 28 | < 0.001 |  |
| 6 | ANASTASSIOU_MULTICANCER_INVASIVENESS_SIGNATURE | 17 | < 0.001 |  |
| 8 | NABA_MATRISOME_ASSOCIATED | 28 | < 0.001 |  |
| 13 | CHARAFE_BREAST_CANCER_LUMINAL_VS_MESENCHYMAL_DN | 22 | < 0.001 |  |
| 14 | REACTOME_EXTRACELLULAR_MATRIX_ORGANIZATION | 19 | < 0.001 |  |
| 18 | VERHAAK_GLIOBLASTOMA_MESENCHYMAL | 16 | < 0.001 |  |
| 34 | NABA_ECM_REGULATORS | 14 | < 0.001 |  |
| 39 | NABA_CORE_MATRISOME | 14 | < 0.001 |  |

Abbreviations: FDR: False Discovery Rate. Genes in overlap indicate number of enriched genes in the corresponding gene set (available at [www.msigdb.org](http://www.msigdb.org)).

|  | Cancer invasion related gene sets |
| --- | --- |
|  | Extracellular matrix related gene sets |
|  | Cell proliferation/differentiation specific gene sets |
|  | Mesenchymal related gene sets |

B. Selected downregulated gene sets from top 50 ranked signalling pathways

| **Rank** | **Gene Set Name** | **Genes in  Overlap** | **FDR (%)** |
| --- | --- | --- | --- |
|  | **C5: *GO gene sets*** |  |  |
| 1 | GO_CILIUM | 22 | < 0.001 |
| 2 | GO_CILIUM_ORGANIZATION | 18 | < 0.001 |
| 3 | GO_MICROTUBULE_ORGANIZING_CENTER | 20 | < 0.001 |
| 4 | GO_MICROTUBULE_CYTOSKELETON | 23 | < 0.001 |
| 5 | GO_CELL_PROJECTION_ASSEMBLY | 17 | < 0.001 |
| 6 | GO_MICROTUBULE_BASED_MOVEMENT | 13 | < 0.001 |
| 8 | GO_CYTOSKELETAL_PART | 25 | < 0.001 |
| 9 | GO_CILIARY_PART | 15 | < 0.001 |
| 10 | GO_MOTILE_CILIUM | 11 | < 0.001 |
| 11 | GO_CELL_PROJECTION_ORGANIZATION | 22 | < 0.001 |
| 12 | GO_MICROTUBULE_BASED_PROCESS | 16 | < 0.001 |
| 13 | GO_CILIUM_MOVEMENT | 7 | < 0.001 |
| 14 | GO_CILIARY_BASAL_BODY | 8 | < 0.001 |
| 15 | GO_CILIUM_OR_FLAGELLUM_DEPENDENT_CELL_MOTILITY | 5 | < 0.001 |
| 16 | GO_CELL_PROJECTION_PART | 17 | < 0.001 |
| 17 | GO_INTRACILIARY_TRANSPORT | 5 | < 0.001 |
| 18 | GO_CILIUM_MOVEMENT_INVOLVED_IN_CELL_MOTILITY | 4 | < 0.001 |
| 19 | GO_EPITHELIAL_CILIUM_MOVEMENT | 4 | < 0.001 |
| 20 | GO_MICROTUBULE_ORGANIZING_CENTER_PART | 7 | < 0.001 |
| 28 | GO_9PLUS2_MOTILE_CILIUM | 5 | 0.002 |
| 36 | GO_CILIARY_TRANSITION_ZONE | 4 | 0.007 |
| 38 | GO_MICROTUBULE_CYTOSKELETON_ORGANIZATION | 8 | 0.020 |
| 40 | GO_MICROTUBULE_BUNDLE_FORMATION | 4 | 0.021 |
| 42 | GO_CILIARY_BASE | 3 | 0.024 |
| 44 | GO_CILIARY_BASAL_BODY_PLASMA_MEMBRANE_DOCKING | 4 | 0.025 |
| 49 | GO_INTRACILIARY_TRANSPORT_INVOLVED_IN_CILIUM_ASSEMBLY | 3 | 0.031 |
|  | **C2: *Curated gene sets*** |  |  |
| 3 | SENGUPTA_NASOPHARYNGEAL_CARCINOMA_DN | 17 | < 0.001 |
| 6 | CHARAFE_BREAST_CANCER_LUMINAL_VS_MESENCHYMAL_UP | 9 | 0.002 |

Abbreviations: FDR: False Discovery Rate. Genes in overlap indicate number of enriched genes in the corresponding gene set (available at [www.msigdb.org](http://www.msigdb.org)).

|  | Cilia related gene sets |
| --- | --- |
|  | Microtubule related gene sets |
|  | Normal tissue specific gene signature |
|  | Non-invasive gene signature |

**Supplementary Figures.**

**Supplementary Figure 1. Contribution of individual variables in prediction modelling of lymph node metastasis in the Bergen training cohort, Model 1.**

**Supplementary Figure 2. Contribution of individual variables in predicting lymph node metastasis in Bergen training cohort, model 2.**


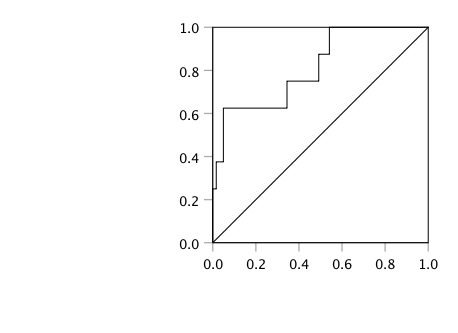

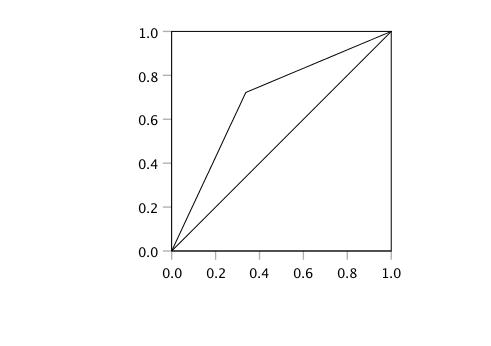


**MRI**

False positive rate

True positive rate

AUC = 0.69

False positive rate

AUC = 0.81

**Fibronectin**


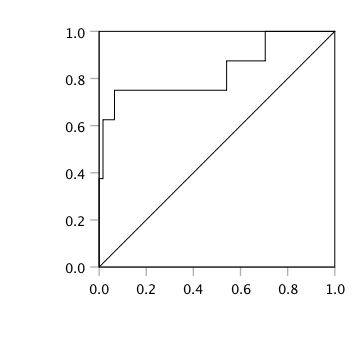


False positive rate

True positive rate

AUC = 0.83

**Combined model**

**A**

**B**

**C**

True positive rate
